# Supplementary material for: Combined MS/MS-NMR Annotation Guided Discovery of Iris lactea var. chinensis Seed as a Source of Viral Neuraminidase Inhibitory Polyphenols
Source: Molecules. 2020 Jul 26;25(15):3383. doi: 10.3390/molecules25153383 (PMC7435927; doi:10.3390/molecules25153383)
Supplement: Supplementary file 1 [file molecules-25-03383-s001.pdf]

Supplementary Materials

Combined MS/MS-NMR Annotation Guided Discovery of *Iris lactea* var. *chinensis* Seed as a Source of Viral Neuraminidase Inhibitory Polyphenols

Hyun Woo Kim [1], Soo Sung Kim [1], Kyo Bin Kang [2], Byeol Ryu [1], Eunjin Park [1], Jungmoo Huh [1], Won Kyung Jeon [3,4], Hee-Sung Chae [1], Won Keun Oh [1], Jinwoong Kim [1], Sang Hyun Sung [1] and Young-Won Chin [1,*]

[1] Research Institute of Pharmaceutical Sciences, College of Pharmacy, Seoul National University, Seoul 08826, Korea; hwkim8906@gmail.com (H.W.K.); soosung179@snu.ac.kr (S.S.K.); estrella56@snu.ac.kr (B.R.); eunjin_p@snu.ac.kr (E.P.); goodhjm112@snu.ac.kr (J.H.); chaeheesung83@gmail.com (H.-S.C.); wkoh1@snu.ac.kr (W.K.O.); jwkim@snu.ac.kr (J.K.); shsung@snu.ac.kr (S.H.S.)

[2] College of Pharmacy, Sookmyung Women's University, Seoul 04310, Korea; kbkang@sookmyung.ac.kr

[3] Herbal Medicine Research Division, Korea Institute of Oriental Medicine, Daejeon 34054, Korea; wkjeon@kist.re.kr

[4] Convergence Research Center for Diagnosis, Treatment and Care System of Dementia, Korea Institute of Science and Technology, Seoul 02792, Korea

* Correspondence: ywchin@snu.ac.kr; Tel.: +82-2-880-7859

Academic Editor: Francesco Cacciola
Received: 29 June 2020; Accepted: 21 July 2020; Published: 26 July 2020

Contents

Figure S1. 1D NMR spectra of compound **1** in methanol-d_4

Figure S2. 2D NMR spectra of compound **1** in methanol-d_4

Figure S3. Sugar analysis data of compounds **1** and **2**

Figure S4. ECD spectrum of compound **1**

Figure S5. 1D NMR spectra of compound **2** in methanol-d_4

Figure S6. 2D NMR spectra of compound **2** in methanol-d_4

Figure S7. ECD spectra of compounds **2** and **7**

Figure S8. HPLC-UV chromatograms of reference compounds

Figure S9. UV absorbance spectra for peak specificity

Figure S10. Inhibitory effect of isolated compounds (**1-10**) on neuraminidase

Figure S11. Inhibitory effect of vitisin C(**10**) on neuraminidase

Figure S12. Shape of *I. lactea* var. *chinensis* seeds

Figure S13. Isolation schemes

Table S1. ^{1}H NMR spectrum data of compounds **1-10**

Table S2. ^{13}C NMR spectrum data of compounds **1-10**

Table S3. A BLASTn analysis result of *matK* sequence of the raw materials.

Table S4. A BLASTn analysis result of *rbcL* sequence of the raw materials.

Table S5. A BLASTn analysis result of *trnLF* sequence of the raw materials.

Figure S1. 1D NMR spectra of compound **1** in methanol-d_4

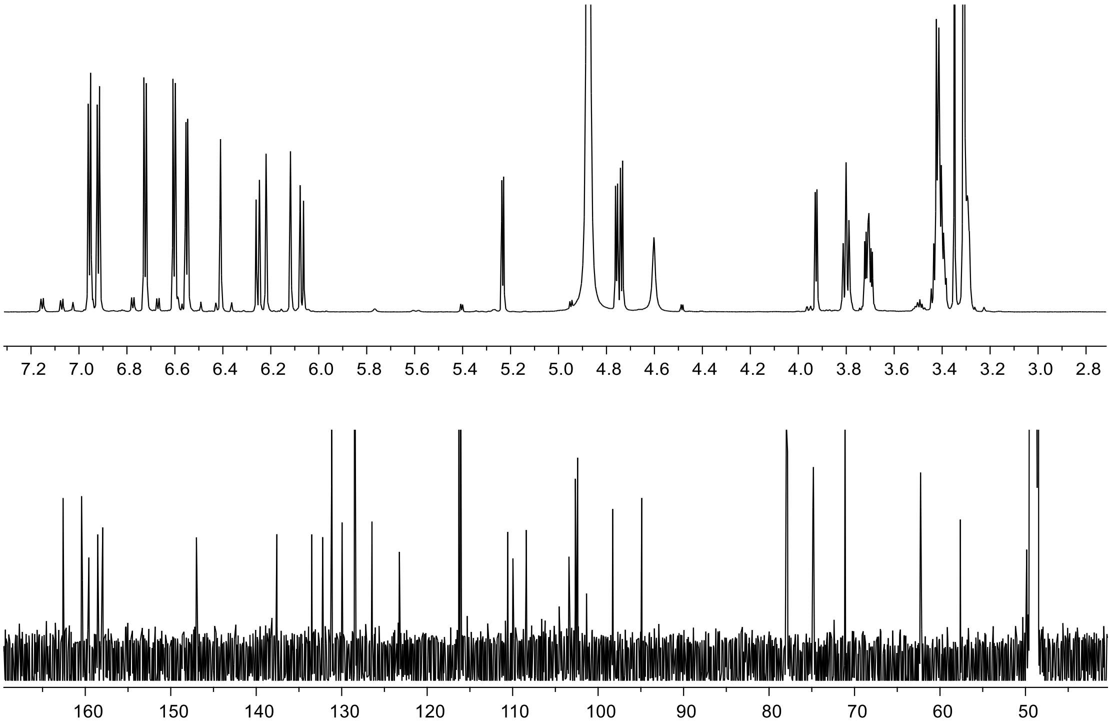

^{1}H and ^{13}C NMR spectra of compound **1**

Figure S2. 2D NMR spectra of compound **1** in methanol-d_4

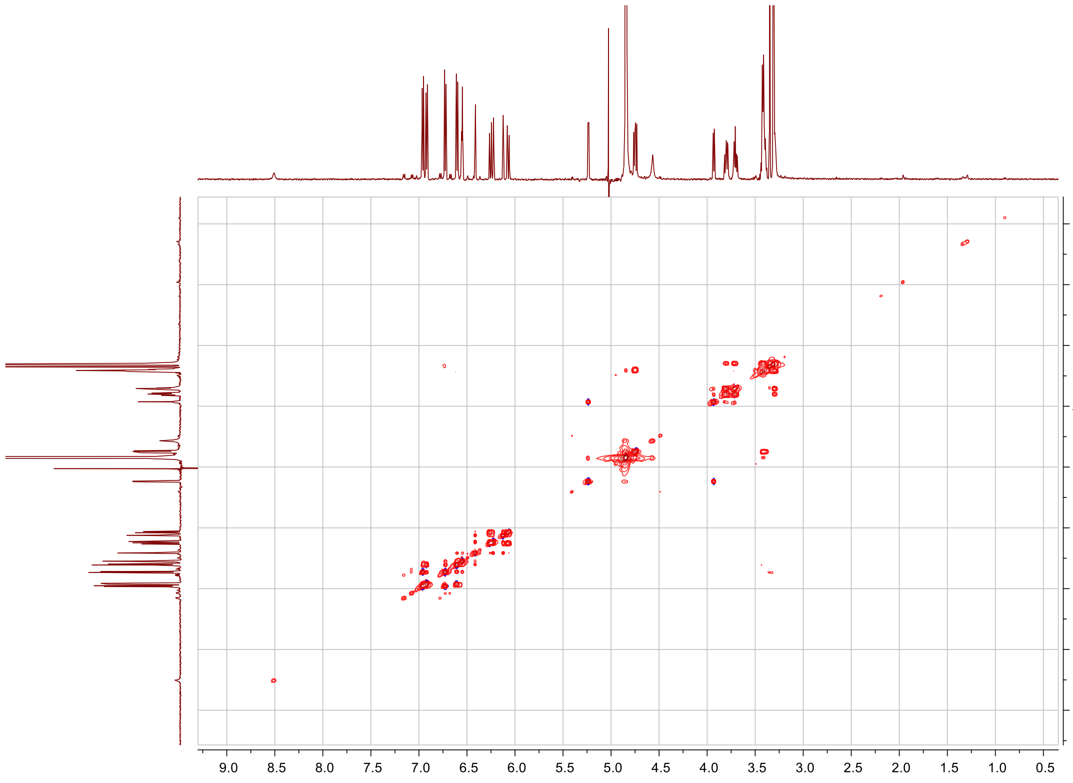

COSY spectrum of compound **1**

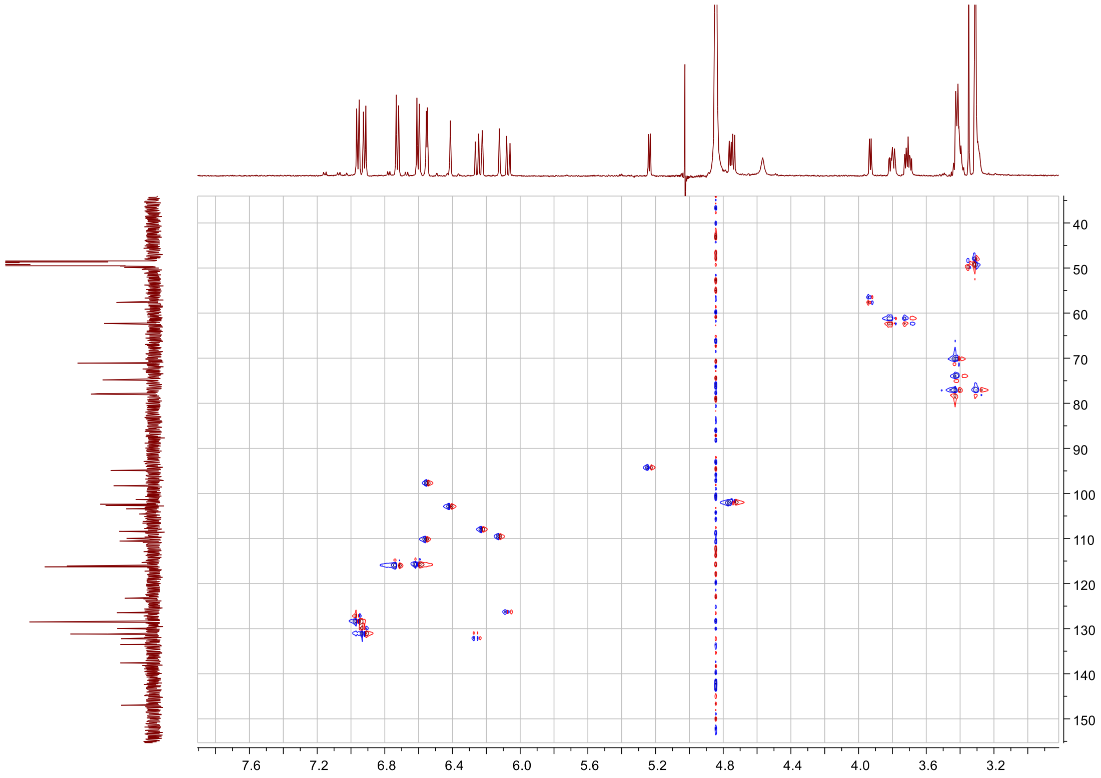

HSQC spectrum of compound **1**

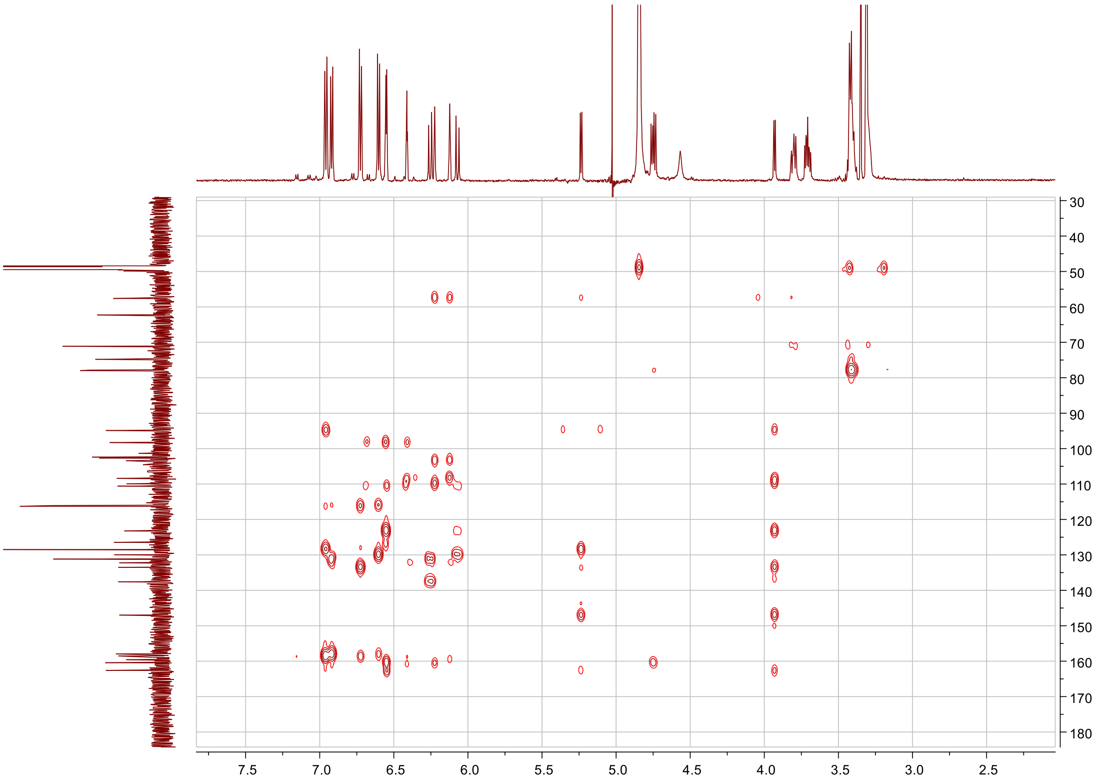

HMBC spectrum of compound **1**

Figure S3. Sugar analysis data of compounds **1** and **2** using HPLC-UV

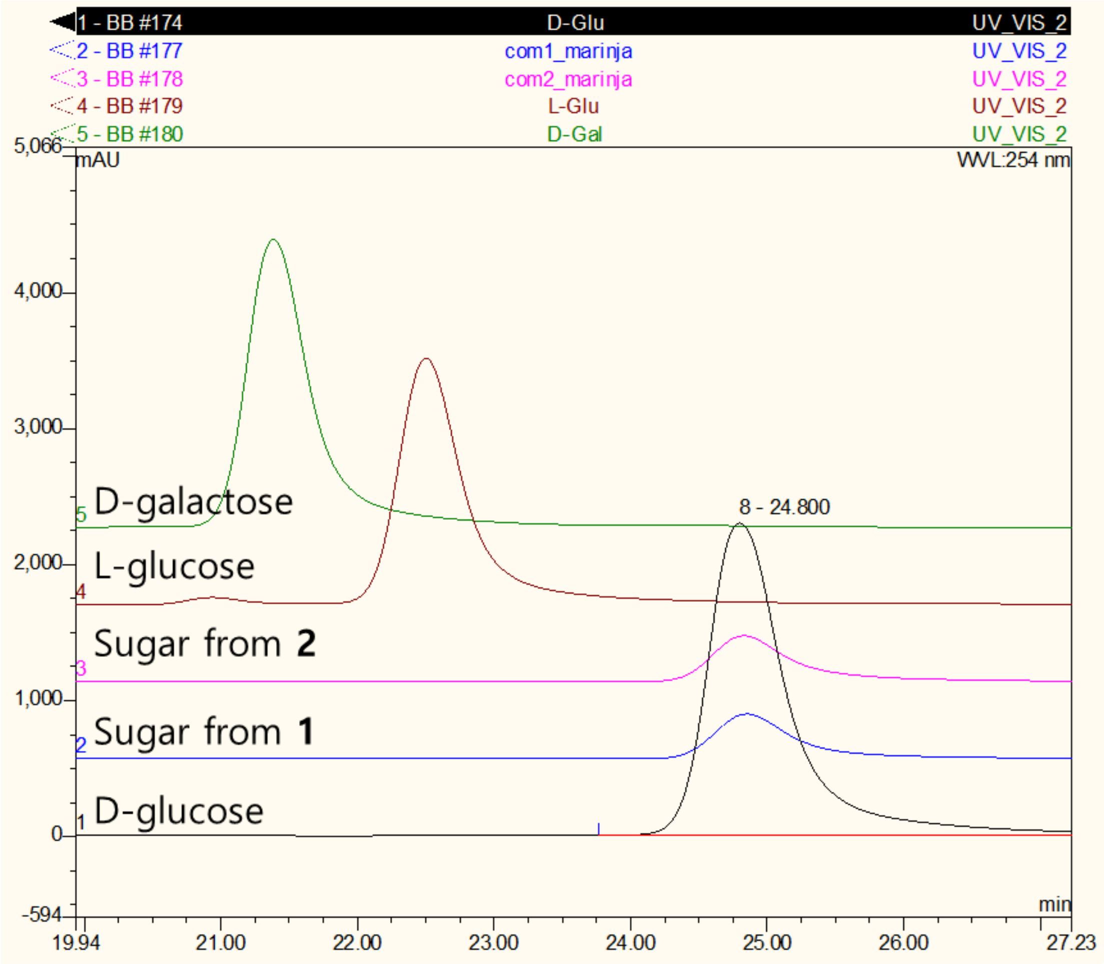

Figure S4. ECD spectrum of compound **1**

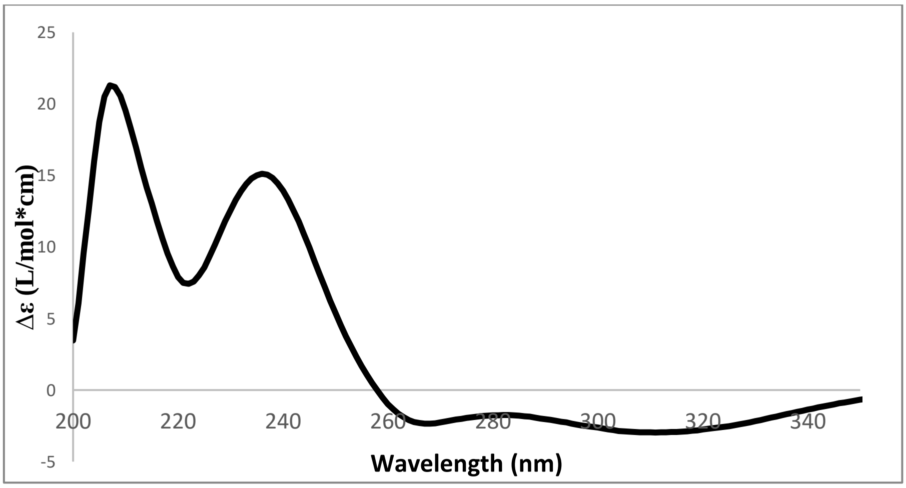

Figure S5. 1D NMR spectra of compound **2** in methanol-d_4

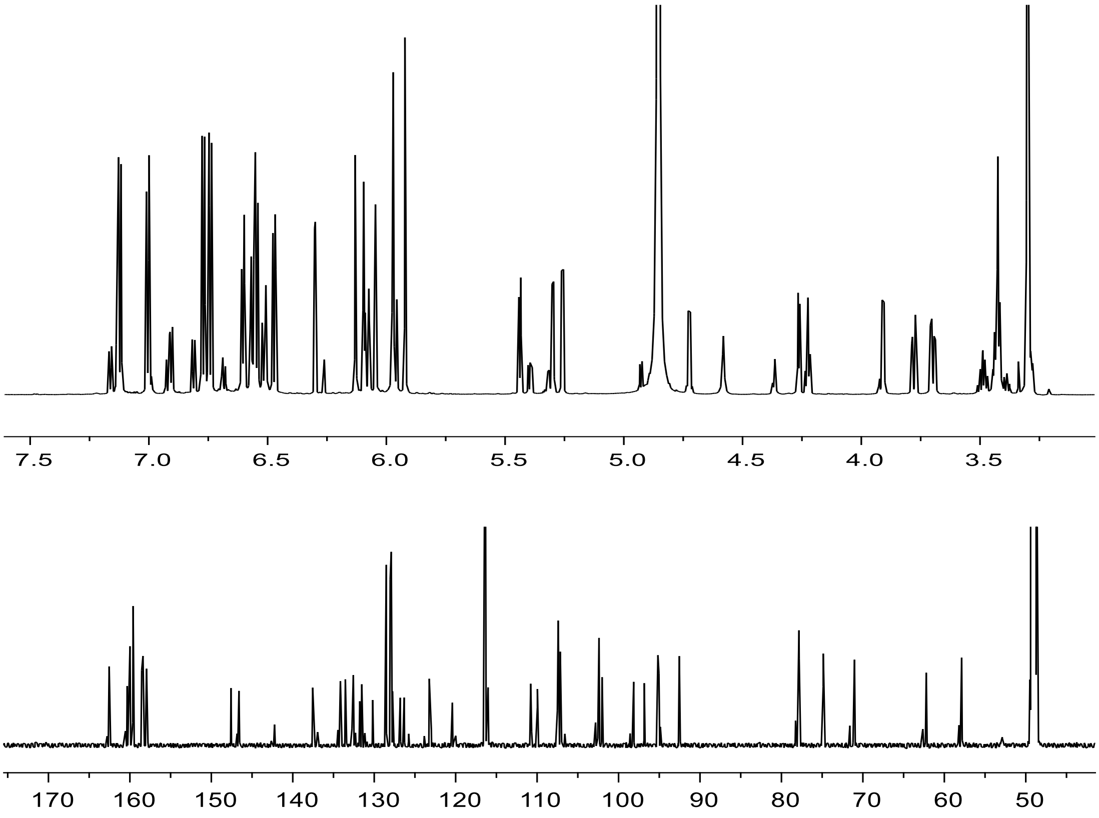

^{1}H and ^{13}C NMR spectra of compound **2**

Figure S6. 2D NMR spectra of compound **2** in methanol-d_4

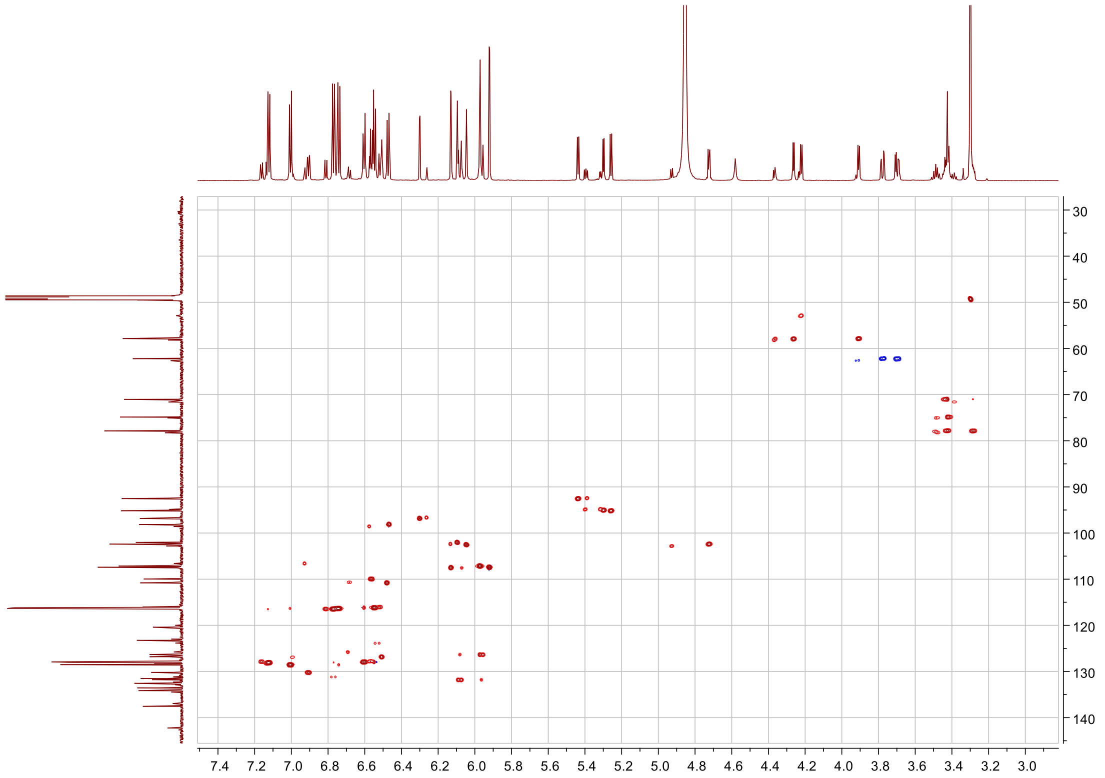

Edited-HSQC spectrum of compound **2**

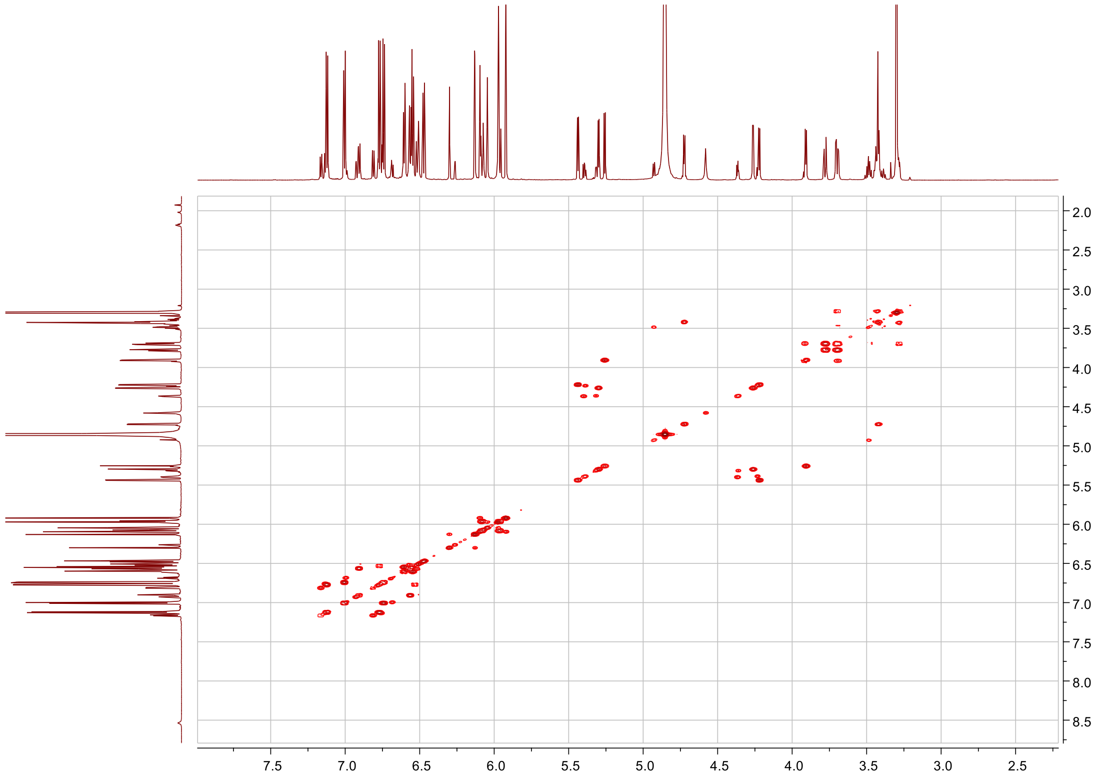

COSY spectrum of compound **2**

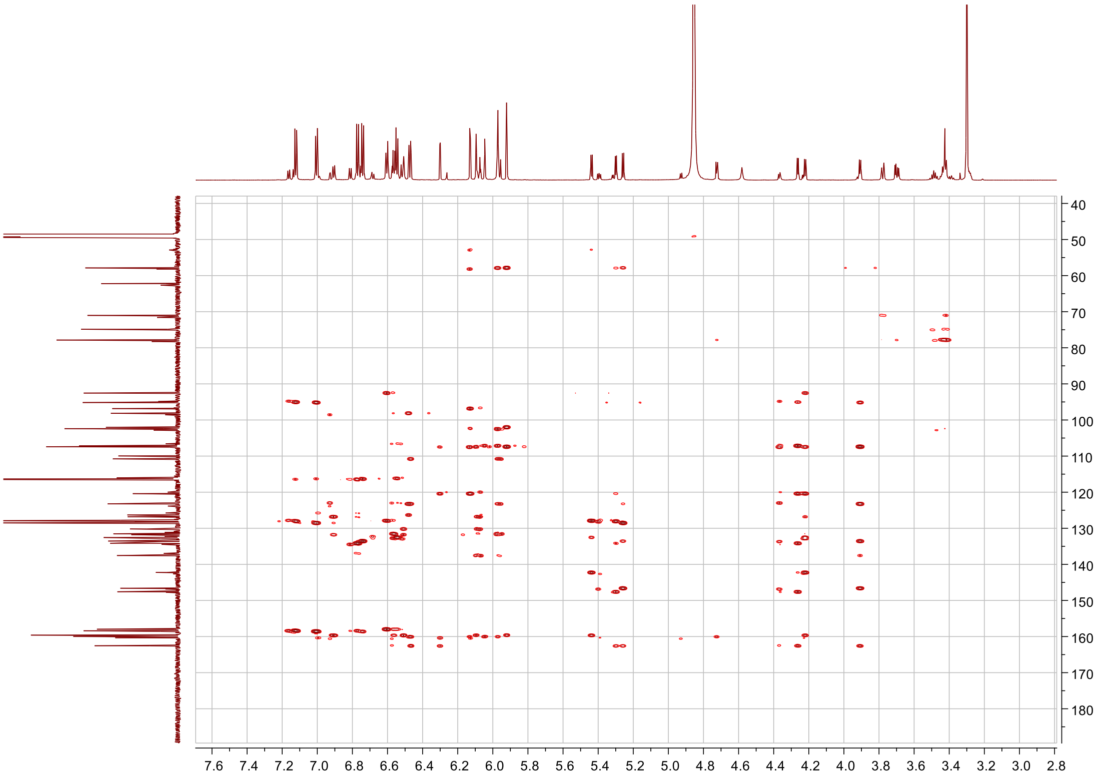

HMBC spectrum of compound **2**

Figure S7. ECD spectra of compounds **2** and **7**

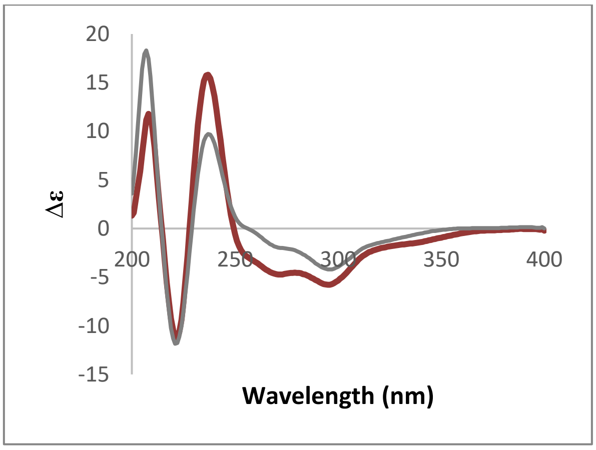

ECD spectra of compounds **2** (brown) and **7** (grey)

Figure S8. HPLC-UV chromatograms of reference compounds

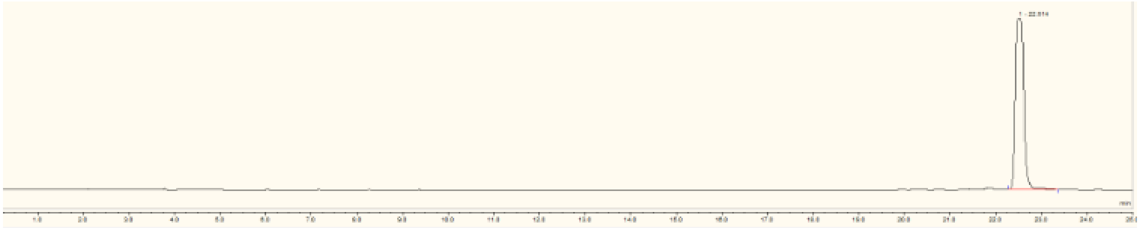

HPLC-UV chromatogram (280 nm) of *trans*-ε-viniferin (**3**)

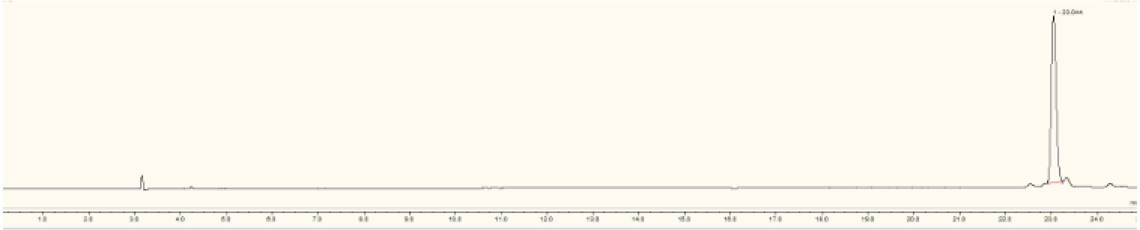

HPLC-UV chromatogram (280 nm) of vitisin A (**6**)

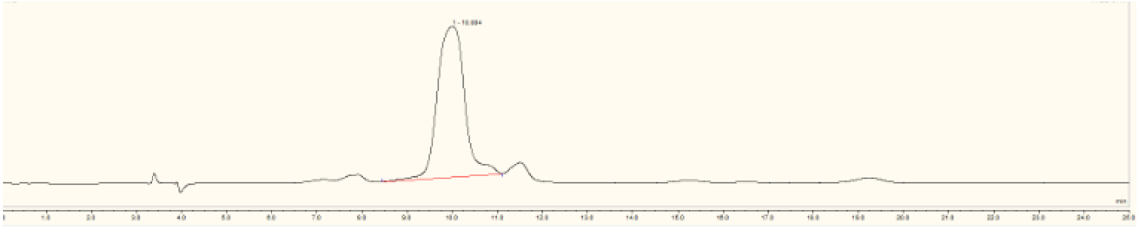

HPLC-UV chromatogram (280 nm) of vitisin B (**9**)

Figure S9. UV absorbance spectra for peak specificity

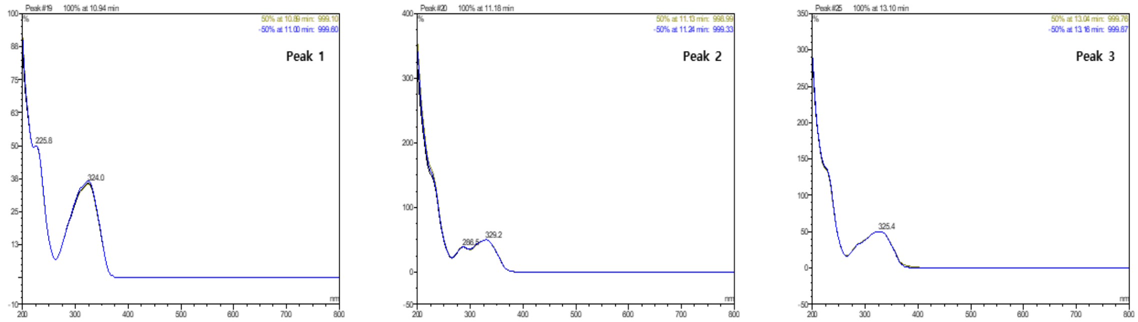

UV absorbance pattern of *trans*-ε-viniferin (peak 1), vitisin A (peak 2), and vitisin B (peak 3) in the sample solution

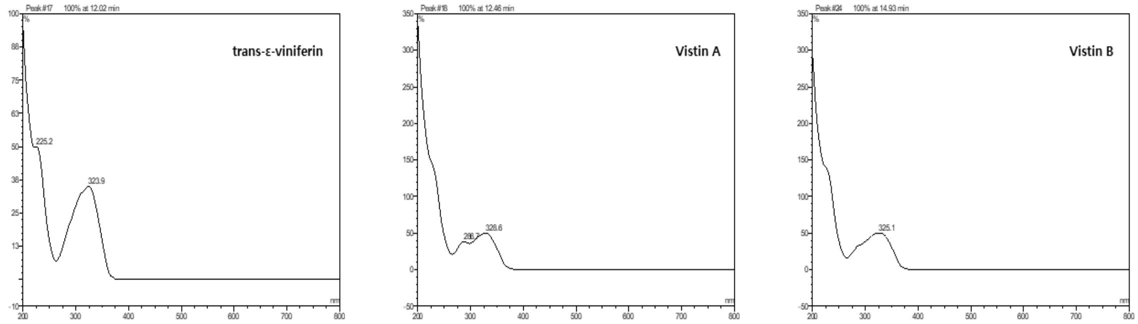

UV absorbance pattern of *trans*-ε-viniferin, vitisin A, and vitisin B from each standard solution

Figure S10. Inhibitory effect of isolated compounds (**1-10**) on neuraminidase

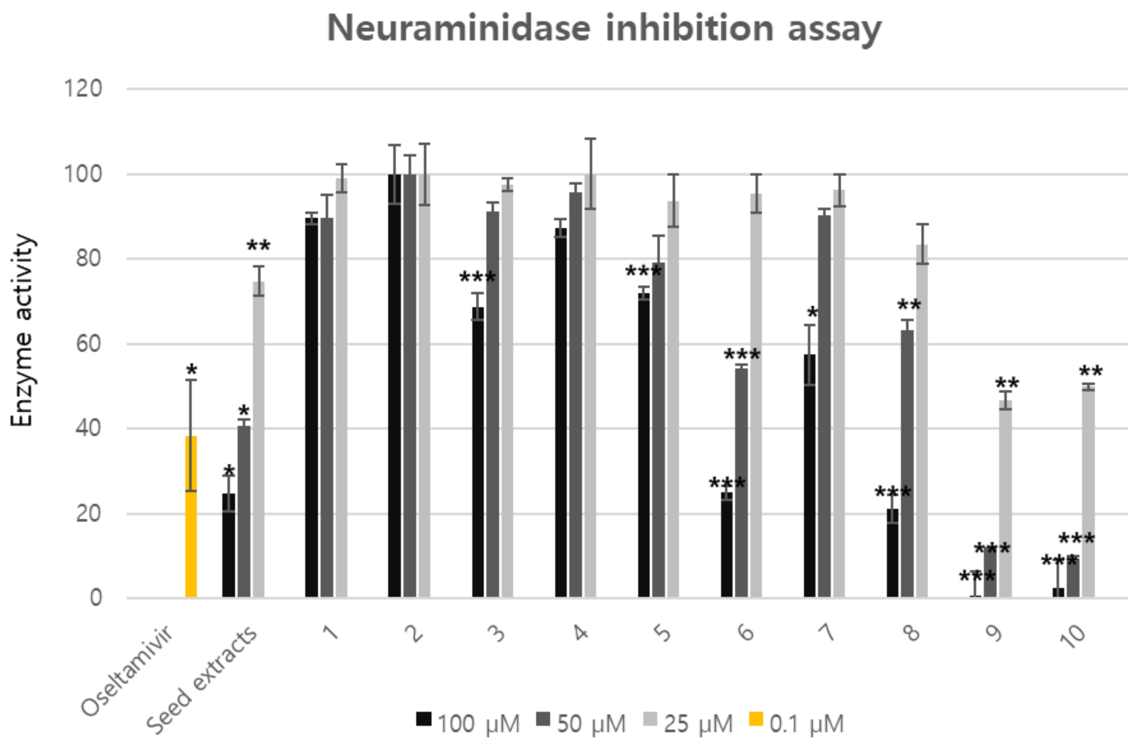

Each value expressed as the mean ± SD ,*p < 0.05, **p < 0.01 and ***p < 0.001 compared to control group. Oseltamivir was used as positive control.

Concentration range of the seed extracts is 100 μg/mL, 50 μg/mL, and 25 μg/mL.

Figure S11. Inhibitory effect of vitisin C(**10**) on neuraminidase

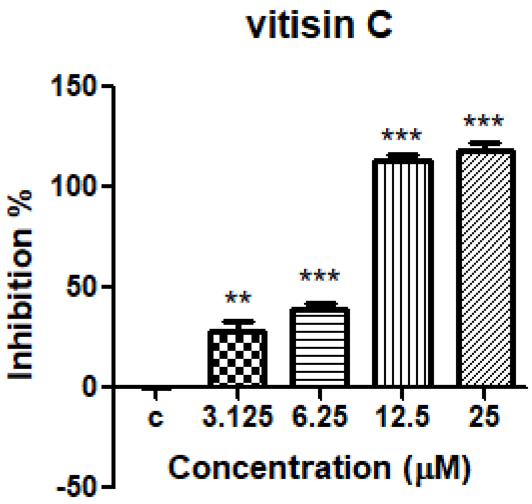

Each value expressed as the mean ± SD (n= 3), *p < 0.05, **p < 0.01 and ***p < 0.001 compared to control group.

Figure S12. Shape of *I. lactea* var. *chinensis* seeds

I. lactea var. *chinensis* seed has an amorphous polyhedron body with dark brown colour.

The length of the seed is around 5 mm and the width is a range of 3 to 4 mm.

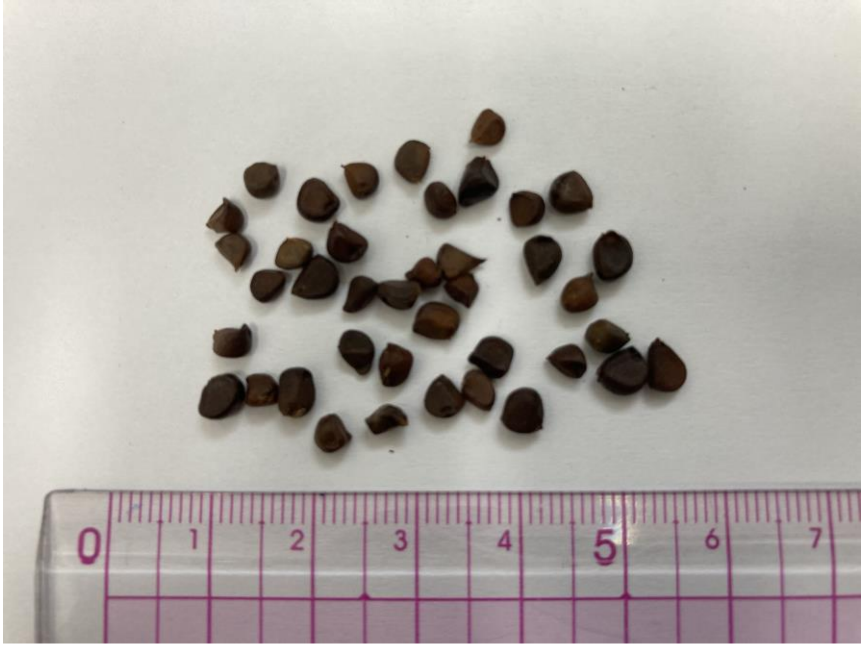

Figure S13. Isolation schemes.

1. Solvent extraction and MS/NMR analysis.

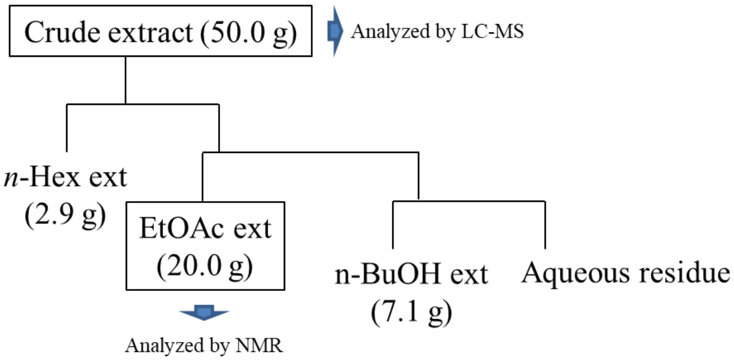

2. Isolation study

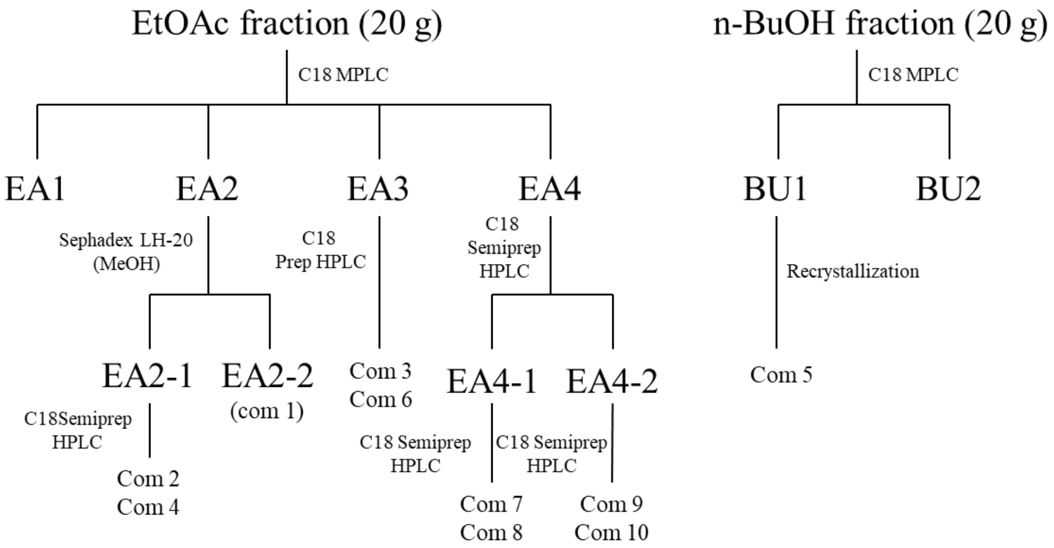

Table S1. ^{1}H NMR spectrum data of compounds **1** to **10**

^{1}H NMR data of compounds **1, 3, 4** and **5** in CD_3OD

position	**1**[a]	**3**[b]	**4**[a]	**5**[a]
1a				
2a/6a	6.96 (d, 8.6)	7.17 (d, 8.5)	7.13 (d, 8.5)	7.17 (d, 8.5)
3a/5a	6.73 (d, 8.6)	6.79 (d, 8.5)	6.76 (d, 8.5)	6.77 (d, 8.5)
7a	5.24 (d, 6.1)	5.39 (d, 6.6)	5.40 (d, 6.7)	5.39 (d, 6.3)
8a	3.33 (d, 6.1)	4.37 (d, 6.6)	4.37 (d, 6.7)	4.48 (d, 6.3)
9a				
10a	6.22 (brs)	6.19 (d, 1.7)	6.15 (d, 2.2)	6.42 (brs)
11a				
12a	6.41 (t, 2.1)	6.21 (t, 1.7)	6.19 (t, 2.2)	6.48 (t, 2.1)
13a				
14a	6.12 (brs)	6.19 (d, 1.7)	6.15 (d, 2.2)	6.35 (d, 1.6)
1b				
2b/6b	6.92 (d, 8.6)	7.06 (d, 8.5)	7.05 (d, 8.6)	7.06 (d, 8.6)
3b/5b	6.60 (d, 8.6)	6.67 (d, 8.5)	6.64 (d, 8.6)	6.66 (d, 8.6)
7b	6.25 (d, 12.0)	6.84 (d, 16.3)	6.91 (d, 16.3)	6.92 (d, 16.4)
8b	6.07 (d, 12.0)	6.59 (d, 16.3)	6.59 (d, 16.3)	6.57 (d, 16.4)
9b				
10b				
11b				
12b	6.54 (d, 1.7)	6.28 (d, 1.4)	6.57 (d, 2.0)	6.58 (d, 1.9)
14b	6.55 (d, 1.7)	6.66 (d, 1.4)	7.01 (d, 2.0)	7.02 (d, 1.9)
13b-Glc				
1′	4.73 (d, 7.1)		4.92 (d, 7.6)	4.72 (d, 7.3)
2′	3.40 - 3.50 (m)		3.40 - 3.50 (m)	3.40 - 3.50 (m)
3′	3.40 - 3.50 (m)		3.40 - 3.50 (m)	3.40 - 3.50 (m)
4′	3.40 - 3.50 (m)		3.40 - 3.50 (m)	3.40 - 3.50 (m)
5′	3.40 - 3.50 (m)		3.40 - 3.50 (m)	3.40 - 3.50 (m)
6′	3.81 (dd, 12.1, 2.2)<br>3.70 (dd, 12.1, 5.1)		3.95 (dd, 12.1, 2.1)<br>3.71 (dd, 12.1, 6.1)	3.95 (dd, 12.1, 2.2)<br>3.70 (dd, 12.1, 5.5)
11a-Glc				
1″	4.76 (d, 7.1)			4.94 (d, 7.3)
2″	3.40 - 3.50 (m)			3.40 - 3.50 (m)
3″	3.40 - 3.50 (m)			3.40 - 3.50 (m)
4″	3.40 - 3.50 (m)			3.40 - 3.50 (m)

5″	3.40 - 3.50 (m)	3.40 - 3.50 (m)
6″	3.84 (dd, 12.1, 2.2)	3.78 (dd, 12.1, 2.2)
	3.65 (dd, 12.1, 5.1)	3.70 (dd, 12.1, 5.5)

[a]Measured in 600 MHz. [b]Mesured in 850 MHz

^{1}H NMR data of compounds **2, 7, 8, 9, 10** in CD_3OD

position	**2**[b]	**7**[a]	**8**[a]	**9**[b]	**10**[b]
1a					
2a/6a	7.00 (d, 8.6)	7.02 (d, 8.5)	6.99 (d, 8.5)	7.14 (d, 8.6)	7.14 (d, 8.6)
3a/5a	6.74 (d, 8.6)	6.75 (d, 8.5)	6.74 (d, 8.5)	6.77 (d, 8.6)	6.76 (d, 8.6)
4a					
7a	5.26 (d, 6.5)	5.22 (d, 6.2)	5.21 (d, 5.5)	5.37 (d, 6.6)	5.35 (d, 6.2)
8a	3.91 (d, 6.5)	3.85 (d, 6.2)	3.76 (d, 5.5)	4.34 (d, 6.6)	4.36 (d, 6.2)
9a					
10a	5.92 (d, 2.2)	5.94 (d, 2.2)	5.99 (d, 2.1)	5.99 (d, 1.6)	5.87 (d, 1.9)
11a					
12a	6.10 (t, 2.1)	6.11 (t, 2.2)	6.10 (t, 2.1)	6.07 (t, 2.1)	6.17 (t, 2.1)
13a					
14a	5.92 (d, 2.2)	5.94 (d, 2.2)	5.99 (d, 2.1)	5.99 (d, 1.6)	5.87 (d, 1.9)
1b					
2b	6.51 (m)	6.54 (d, 2.2)	6.51 (brs)	6.67 (m)	6.59 (d, 1.6)
3b					
4b					
5b	6.56 (d, 8.3)	6.56 (d, 8.3)	6.45 (d, 8.3)	6.68 (d, 8.6)	6.63 (d, 8.4)
6b	6.91 (dd, 8.3, 1.3)	6.92 (dd, 8.3, 1.1)	6.83 (brd, 8.3)	6.99 (dd, 8.4, 1.5)	6.99 (dd, 8.4, 1.5)
7b	6.08 (d, 12.2)	6.07 (d, 12.2)	6.17 (d, 12.0)	6.52 (d, 17.0)	6.73 (d,16.3)
8b	5.96 (d, 12.2)	5.97 (d, 12.2)	6.02 (d, 12.0)	6.69 (d, 17.0)	6.55 (d,16.3)
9b					
10b					
11b					
12b	6.47 (d, 2.1)	6.19 (d, 2.1)	6.24 (brs)	6.23 (d, 2.1)	6.25 (brs)
13b					
14b	6.48 (d, 2.1)	6.21 (d, 2.1)	6.24 (brs)	6.11 (d, 2.1)	6.13 (d, 1.9)
1c					
2c/6c	6.60 (d, 8.6)	6.61 (d, 8.5)	7.01 (d, 8.5)	6.59 (d, 8.5)	6.96 (d, 8.5)
3c/5c	6.55 (d, 8.6)	6.56 (d, 8.5)	6.68 (d, 8.5)	6.53 (d, 8.5)	6.66 (d, 8.5)
4c					
7c	5.44 (d, 5.8)	5.45 (d, 5.6)	5.09 (d, 10.5)	5.43 (d, 5.0)	5.17 (d, 9.8)
8c	4.22 (d, 5.8)	4.23 (d, 5.6)	4.23 (d, 10.5)	4.26 (d, 5.0)	4.27 (d, 9.8)
9c					
10c					

11c					
12c	6.30 (t, 2.1)	6.30 (d, 2.1)	6.26 (d, 2.1)	6.26 (d, 2.1)	6.24 (brs)
13c					
14c	6.13 (d, 2.1)	6.12 (d, 2.1)	6.06 (d, 2.1)	6.59 (d, 1.9)	6.60 (d, 1.6)
1d					
2d/6d	7.12 (d, 8.4)	7.13 (d, 8.6)	6.89 (d, 8.5)	7.19 (d, 8.6)	6.94 (d, 8.6)
3d/5d	6.77 (d, 8.4)	6.78 (d, 8.6)	6.73 (d, 8.5)	6.83 (d, 8.6)	6.74 (d, 8.6)
4d					
7d	5.30 (d, 5.2)	5.31 (d, 5.0)	5.12 (d, 5.1)	5.34 (d, 4.5)	5.19 (d, 5.0)
8d	4.26 (d, 5.2)	4.28 (d, 5.0)	3.47 (d, 5.1)	4.37 (d, 4.5)	3.59 (d, 5.0)
9d					
10d	5.97 (brs)	5.99 (d, 1.8)	5.83 (d, 2.1)	6.15 (d, 2.1)	6.14 (d, 2.1)
11d					
12d	6.05 (t, 2.1)	6.09 (d, 2.1)	6.06 (d, 2.1)	6.14 (d, 2.1)	6.10 (d, 2.1)
13d					
14d	5.97 (brs)	5.99 (d, 1.8)	5.83 (d, 2.1)	6.15 (d, 2.1)	6.14 (d, 2.1)
13b-Glc					
1′	4.72 (d, 7.2)				
2′	3.30 - 3.50 (m)				
3′	3.30 - 3.50 (m)				
4′	3.30 - 3.50 (m)				
5′	3.30 - 3.50 (m)				
6′	3.78 (dd, 12.2, 2.3)<br>3.70 (dd, 12.2, 4.8)				

[a]Measured in 600 MHz. [b]Mesured in 850 MHz

Table S2. ^{13}C NMR spectrum data of compounds **1 - 10**

^{13}C NMR data of compounds **1, 3, 4** and **5** in CD_3OD (δ in ppm)

position	**1**[a]	**3**[b]	**4**[a]	**5**[a]
1a	133.5	132.5	133.6	134.5
2a/6a	128.5	127.4	128.2	129.0
3a/5a	116.3	115.0	116.3	117.2
4a	158.5	157.1	158.6	159.5
7a	94.9	93.4	94.9	95.7
8a	57.6	56.9	58.2	58.7
9a	147.0	146.0	146.9	147.8
10a	108.4	106.1	107.5	109.7
11a	160.3	158.7	160.1	161.7
12a	103.4	100.8	102.9	104.6
13a	159.6	158.7	160.1	160.8
14a	110.0	106.1	107.5	111.0
1b	130.0	129.0	130.3	131.0
2b/6b	131.2	126.8	128.9	129.8
3b/5b	116.1	114.9	116.4	117.3
4b	157.9	157.0	158.5	159.4
7b	132.2	129.0	131.0	132.1
8b	126.4	122.3	123.2	124.0
9b	137.5	135.5	137.0	137.9
10b	123.2	118.7	122.9	123.4
11b	162.6	161.4	162.5	163.4
12b	98.3	95.5	98.5	99.3
13b	160.4	158.4	160.6	161.5
14b	110.6	103.0	106.1	107.1
13b-Glc				
1′	102.4		102.3	103.4
2′	74.8		75.0	75.8
3′	77.9		78.0	78.8
4′	71.1		71.5	72.4
5′	78.0		78.3	79.2
6′	62.3		62.6	63.4
11a-Glc				
1″	102.6			103.6
2″	74.8			75.6

3″	77.8				78.8
4″	71.1				71.9
5″	78				78.86
6″	62.2				63.08

[a]measured in 213 MHz. [b]measured in 150 MHz.

^{13}C NMR data of compounds **2, 7, 8, 9** and **10** in CD_3OD (δ in ppm)

position	**2**[a]	**7**[b]	**8**[b]	**9**[b]	**10**[a]
1a	133.5	133.9	134.5	133.9	134.0
2a	128.5	128.5	128.0	128.2	128.2
3a	116.3	116.3	116.4	116.3	116.3
4a	158.6	158.4	158.3	158.5	158.4
5a	116.3	116.3	116.4	116.3	116.3
6a	128.5	128.5	128.0	128.2	128.2
7a	95.2	94.9	94.6	94.8	94.8
8a	57.9	57.8	58.1	58.2	58.2
9a	146.6	147.2	147.6	147.2	147.7
10a	107.4	107.3	107.2	107.0	107.2
11a	159.6	159.5	159.6	160.0	160.0
12a	102.2	101.9	101.9	102.5	102.2
13a	159.6	159.5	159.6	160.0	160.0
14a	107.4	107.3	107.2	107.0	107.2
1b	131.5	131.6	131.8	132.3	132.2
2b	126.8	126.9	126.7	125.5	125.8
3b	127.9	132.5	131.2	128.5	131.6
4b	159.6	159.5	160.6	160.2	161.2
5b	110.0	109.9	109.8	110.7	110.4
6b	130.2	130.0	129.9	126.7	126.6
7b	131.8	131.4	131.9	130.5	130.7
8b	126.3	126.7	127.0	124.2	124.3
9b	137.6	137.5	137.7	136.8	137.0
10b	123.3	120.3	120.2	120.1	121.9
11b	162.6	162.7	163.1	162.8	162.8
12b	98.1	96.8	96.7	96.6	96.9
13b	160.0	159.4	159.4	160.5	159.7
14b	110.8	108.8	109.0	107.5	104.9
1c	132.6	142.3	131.8	142.5	132.3
2c	127.9	127.8	129.2	127.9	128.8
3c	116.2	116.1	116.3	116.0	116.4

4c	158.0	157.9	158.8	158.0	158.8
5c	116.2	116.1	116.3	116.0	116.4
6c	127.9	127.8	129.2	127.9	128.8
7c	92.5	92.2	95.0	92.2	95.0
8c	52.9	52.9	55.4	52.9	55.2
9c	142.3	132.7	148.2	132.7	140.8
10c	120.4	120.3	122.2	120.0	119.9
11c	162.5	162.6	162.4	162.7	162.6
12c	96.8	96.7	96.9	96.9	96.9
13c	160.3	160.4	160.1	159.6	160.4
14c	107.5	107.3	108.7	104.6	108.2
1d	134.2	134.2	133.9	134.6	133.4
2d	128.0	127.9	128.2	127.8	128.1
3d	116.5	116.4	116.3	116.5	116.3
4d	158.5	158.4	158.4	158.4	158.5
5d	116.5	116.4	116.3	116.5	116.3
6d	128.0	127.9	128.2	127.8	128.1
7d	95.0	94.9	94.6	94.7	94.5
8d	57.8	57.9	56.7	57.9	56.3
9d	147.6	147.6	147.6	147.8	147.5
10d	107.2	107.1	107.6	107.5	107.4
11d	159.9	160.0	159.8	160.1	159.9
12d	102.5	102.5	102.2	102.3	102.2
13d	159.9	160.0	159.8	160.1	159.9
14d	107.2	107.1	107.6	107.5	107.4
1′	102.4				
2′	74.9				
3′	77.8				
4′	71.0				
5′	77.8				
6′	62.2				

[a]measured in 213 MHz. [b]measured in 150 MHz.

Table S3. BLASTn analysis of *matK* sequence of the raw materials.

Description	Max Score	Total Score	Query Cover	E value	Per. Ident	Accession
Iris lactea voucher ZhouSL-shanxi-Z033 ribulose-1,5-bisphosphate carboxylase/oxygenase large subunit (rbcL) gene, partial cds; chloroplast	881	881	100%	0	100.00%	JF942062.1
Iris lactea isolate Wenchuan ribulose-1,5-bisphosphate carboxylase/oxygenase large subunit gene, partial cds; chloroplast	865	865	100%	0	99.37%	KX518311.1
Iris lactea var. lactea voucher z040 ribulose-1,5-bisphosphate carboxylase/oxygenase large subunit (rbcL) gene, partial cds; chloroplast	865	865	100%	0	99.37%	KP089570.1
Iris lactea voucher ZhouSL-zhuoni-Z040 ribulose-1,5-bisphosphate carboxylase/oxygenase large subunit (rbcL) gene, partial cds; chloroplast	865	865	100%	0	99.37%	JF942061.1
Iris lactea voucher ZhouSL-sanggendalai-Z214 ribulose-1,5-bisphosphate carboxylase/oxygenase large subunit (rbcL) gene, partial cds; chloroplast	859	859	100%	0	99.16%	JF942063.1
Iris bloudowii ribulose-1,5-bisphosphate carboxylase/oxygenase large subunit (rbcL) gene,	843	843	100%	0	98.53%	MF158721.1
Iris setosa voucher CCDB-18343-C4 ribulose-1,5-bisphosphate carboxylase/oxygenase large subunit (rbcL) gene, partial cds; chloroplast	843	843	100%	0	98.53%	MG228050.1
Iris setosa voucher CCDB-18343-D4 ribulose-1,5-bisphosphate carboxylase/oxygenase large subunit (rbcL) gene, partial cds; chloroplast	843	843	100%	0	98.53%	MG227615.1
Iris brevicaulis voucher CCDB-18296-B01 ribulose-1,5-bisphosphate carboxylase/oxygenase large subunit (rbcL) gene, partial cds; chloroplast	843	843	100%	0	98.53%	MG227367.1
Iris ensata voucher CCDB-24917-E01 ribulose-1,5-bisphosphate carboxylase/oxygenase large subunit (rbcL) gene, partial cds; chloroplast	843	843	100%	0	98.53%	MG226738.1

Table S4. BLASTn analysis of *rbcL* sequence of the raw materials.

Description	Max Score	Total Score	Query Cover	E value	Per. Ident	Accession
Iris oxypetala chloroplast DNA containing partial trnL gene, trnL-trnF IGS and partial trnF gene, isolate QAN	1086	1086	100%	0	100.00%	LT627914.1
Iris oxypetala chloroplast DNA containing partial trnL gene, trnL-trnF IGS and partial trnF gene, isolate ALM	1086	1086	100%	0	100.00%	LT627905.1
Iris oxypetala chloroplast tRNA-Leu gene (partial), trnL-trnF IGS and tRNA-Phe gene (partial), isolate POS	1086	1086	100%	0	100.00%	LN871606.1
Iris lactea clone NEFUIris0004 tRNA-Leu (trnL) gene and trnL-trnF intergenic spacer, partial sequence; chloroplast	1086	1086	100%	0	100.00%	DQ286787.1
Iris lactea chloroplast DNA containing partial trnL gene, trnL-trnF IGS and partial trnF gene, isolate XIN	1081	1081	100%	0	99.83%	LT627929.1
Iris oxypetala chloroplast tRNA-Leu gene (partial), trnL-trnF IGS and tRNA-Phe gene (partial), haplotype B, isolate USS	1077	1077	100%	0	99.66%	LN871608.1
Iris lactea chloroplast DNA containing partial trnL gene, trnL-trnF IGS and partial trnF gene, isolate BEI_2	1075	1075	100%	0	99.66%	LT627935.1
Iris lactea chloroplast DNA containing partial trnL gene, trnL-trnF IGS and partial trnF gene, isolate BAY	1075	1075	100%	0	99.66%	LT627924.1
Iris lactea chloroplast DNA containing partial trnL gene, trnL-trnF IGS and partial trnF gene, isolate IMH	1075	1075	100%	0	99.66%	LT627918.1
Iris lactea chloroplast tRNA-Leu gene (partial), trnL-trnF IGS and tRNA-Phe gene (partial), isolate ORK	1075	1075	100%	0	99.66%	LN871624.1

Table S5. BLASTn analysis of *trnLF* sequence of raw materials.

Description	Max Score	Total Score	Query Cover	E value	Per. Ident	Accession
Iris oxypetala chloroplast DNA containing partial trnL gene, trnL-trnF IGS and partial trnF gene, isolate QAN	1086	1086	100%	0	100.00%	LT627914.1
Iris oxypetala chloroplast DNA containing partial trnL gene, trnL-trnF IGS and partial trnF gene, isolate ALM	1086	1086	100%	0	100.00%	LT627905.1
Iris oxypetala chloroplast tRNA-Leu gene (partial), trnL-trnF IGS and tRNA-Phe gene (partial), isolate POS	1086	1086	100%	0	100.00%	LN871606.1
Iris lactea clone NEFUIris0004 tRNA-Leu (trnL) gene and trnL-trnF intergenic spacer, partial sequence; chloroplast	1086	1086	100%	0	100.00%	DQ286787.1
Iris lactea chloroplast DNA containing partial trnL gene, trnL-trnF IGS and partial trnF gene, isolate XIN	1081	1081	100%	0	99.83%	LT627929.1
Iris oxypetala chloroplast tRNA-Leu gene (partial), trnL-trnF IGS and tRNA-Phe gene (partial), haplotype B, isolate USS	1077	1077	100%	0	99.66%	LN871608.1
Iris lactea chloroplast DNA containing partial trnL gene, trnL-trnF IGS and partial trnF gene, isolate BEI_2	1075	1075	100%	0	99.66%	LT627935.1
Iris lactea chloroplast DNA containing partial trnL gene, trnL-trnF IGS and partial trnF gene, isolate BAY	1075	1075	100%	0	99.66%	LT627924.1
Iris lactea chloroplast DNA containing partial trnL gene, trnL-trnF IGS and partial trnF gene, isolate IMH	1075	1075	100%	0	99.66%	LT627918.1
Iris lactea chloroplast tRNA-Leu gene (partial), trnL-trnF IGS and tRNA-Phe gene (partial), isolate ORK	1075	1075	100%	0	99.66%	LN871624.1
